# The Prevalence of Periodontitis and Assessment of Oral Micro-Biota in Patients with Hidradenitis Suppurativa: A Descriptive Cross-Sectional Study

**DOI:** 10.3390/jcm11237065

**Published:** 2022-11-29

**Authors:** Beata Jastrząb, Barbara Paśnik-Chwalik, Tomasz Konopka, Piotr K. Krajewski, Jacek C. Szepietowski, Łukasz Matusiak

**Affiliations:** 1 Department of Dermatology, Venereology and Allergology, Wroclaw Medical University, 50-368 Wroclaw, Poland; 2 Department of Periodontology, Wroclaw Medical University, 50-425 Wroclaw, Poland

**Keywords:** hidradenitis suppurativa, periodontitis, oral health, oral microbiota

## Abstract

Periodontitis has been causally connected with the development of other immune-mediated inflammatory disorders previously. Nevertheless, the current literature does not provide knowledge on oral health in hidradenitis suppurativa (HS) individuals. The aim of this study was to assess the prevalence of periodontitis and characterize an oral microbiome in HS patients. Fifty-five patients with HS and fifty-five healthy controls were enlisted in the study. The incidence of periodontitis was assessed in all patients during the periodontal evaluation. RT-PCR tests were used to quantification of bacterial content and assess the number and composition of nine crucial periodontal pathogens. HS patients had a significantly higher prevalence of periodontitis than healthy controls (45.5% versus 14.5%). Significantly higher values of average copy-count numbers of total bacteria were found in HS patients. The majority of periodontal pathogens were more frequently isolated in patients with HS than among controls. The most frequently detected pathogen in the HS group was *Treponema denticola* (70.9%), whereas among controls *Capnocytophaga gingivalis* (34.5%) was the most common isolate. There was no correlation between HS severity and the number of DNA copies of periodontal bacteria. The findings of this research suggest that periodontitis may contribute to the development of HS.

## 1. Introduction

Hidradenitis suppurativa (HS) is a chronic inflammatory disorder of the pilosebaceous unit of the intertriginous body regions, characterized by recurrent nodules, abscesses, and tunnels [1]. The greater risk of manifesting HS extends from late adolescence to the fourth decade of life with a female preponderance [2,3]. The etiology is multifaceted and seems to be a synergistic relationship between impaired innate immunity and genetics, hormonal, lifestyle, and microbial factors [4].

Periodontitis is a chronic, inflammatory disease of the tooth-supporting structures that may result in the destruction of alveolar bone and periodontal ligament, leading to tooth loss [5]. The disorder has been reported to affect almost 50% of the adult population in the western world, with the most severe form occurring in 11.2% of the global population [6,7]. The pathogenic mechanisms of the disease involve complex interlinkages among infection with anaerobic bacteria in periodontal pockets, excessive host immune responses, and environmental factors, including smoking [5,8]. Microbial infection in subgingival plaque biofilm with periopathogens (bacterial species that contribute to periodontitis) leads to chronic inflammation in vulnerable individuals [9,10]. Socransky et al. [9] classified oral microbes into several groups (complexes) based on microorganism correlations and their involvement in the etiology of periodontal disorders. The most crucial for periodontal tissues and regarded as a pathogenic consortium in periodontitis is the red complex, which comprises the following species: *Porphyromonas gingivalis*, *Tannerella forsythia,* and *Treponema denticola*. This complex manifests the strongest correlation with the clinical parameters considered most significant in periodontal diseases such as pocket depth and bleeding on probing [9,11,12]. The orange complex, closely related to the red one, contains *Prevotella intermedia*, *Peptostreptococcus micros*, *Fusobacterium nucleatum,* and *Campylobacter rectus*. The green complex includes *Capnocytophaga gingivalis*, *Campylobacter concisus*, *Eikenella corrodens*, and *Aggregatibacter actinomycetemcomitans* serotype a. The yellow complex consists of various *Streptococci.* Periopathogens such as *Aggregatibacter actinomycetemcomitans* serotype b, *Actinomyces naeslundii genospecies,* and *Selenomonas noxia* are separate microorganisms, and they do not cluster with other species [9].

The staging guidelines of periodontitis divide the classification into staging and grading of the disease. Staging is a measure used to assess the severity and extent of the management required (stage I: initial periodontitis; stage II: moderate periodontitis; stages III and IV: severe periodontitis), whereas grading is based on evidence of progression rate in three categories: slow (grade A), moderate (grade B) and rapid rate of progression (grade C) [5].

Periodontitis has been causally connected with the development of other immune-mediated inflammatory disorders (IMIDs), such as psoriasis, psoriatic arthritis, rheumatoid arthritis, and inflammatory bowel diseases. The dysbiotic biofilm associated with periodontal disease may contribute to these disorders directly or via enhancing the immunological system [13,14,15,16,17]. Although studies on oral health in HS patients are limited and the primary etiology of HS and periodontitis appears different, both diseases seem to share several pathogenetic features [18,19,20,21,22]. Confirming this relationship is important and could result in early periodontal management and prevent teeth loss, as well as may provide a good starting point for further investigation on unraveling new pathogenetic pathways common to both disorders.

The primary purpose of this study was to characterize an oral microbiome in patients with HS in comparison to healthy controls and investigate the potential association between HS and periodontitis.

## 2. Materials and Methods

### 2.1. Study Population

Between December 2021 and May 2022, 55 consecutive adult HS individuals (27 females and 28 males, aged 19–61 years, mean age = 36.22, SD = 10.97) attending the outpatient unit at the Department of Dermatology, Venereology and Allergology of Wroclaw University Hospital, were enlisted to the study. The corresponding number of healthy controls was matched with HS patients in terms of age and gender. Before the study commencement, the protocol was accepted by the local ethical committee (Consent no. 919/2021, date: 26 November 2021). The study was conducted according to the principles of Helsinki’s Declaration, and a written participation agreement was obtained from each study subject after elucidating the purpose and nature of the investigation. The exclusion criteria for this study were the following: being under the age of 18, pregnancy, breastfeeding, and use of any systemic antimicrobials within 3 months prior to study entry.

### 2.2. Clinical Evaluation

Detailed information on demographics, lifestyle, comorbidities, and previous treatment in both groups was collected and analyzed. HS severity stage was assessed in all patients during the dermatological evaluation using the Hurley staging system, and the clinical severity of HS was determined with International Hidradenitis Suppurativa Severity Score System (IHS4) [23,24,25].

The diagnosis of periodontitis was made in accordance with the new classification and case definition of periodontitis revised in 2018 [26]. Participants from study and control groups were examined by a periodontist and identified as periodontitis cases in the context of clinical care if interdental clinical attachment loss (CAL) was detectable at two or more non-adjacent teeth or if buccal or oral CAL no less than 3 mm with pocketing more than 3 mm was detectable at two or more teeth [26].

### 2.3. Sample Collection and Processing

The deepest periodontal pocket was selected for examination, and gingival sulcus samples were collected from every study subject. Prior to the procedure, supragingival bacterial plaque was removed, and the examined sites were carefully dried using sterile cotton swaps. Next, with sterile tweezers, the paper point from the diagnostic kit was inserted full depth in periodontal pockets for 20 seconds. In the event of bleeding, collecting samples was repeated.

The samples were packed into labeled test tubes, located in a transportation set, and sent to a MIP Pharma Laboratory for bacterial culture and count.

### 2.4. Bacterial Identification

Microbiological analysis was accomplished by using a real-time polymerase chain reaction (RT-PCR). Diagnostic kit PET Test^®^ plus (MIP Pharma, Blieskastel, Germany) was used for quantification of bacterial content and to assess the number and composition of nine following periopathogens: *Porphyromonas gingivalis, Treponema denticola, Tannerella forsythia* from the red complex, *Prevotella intermedia, Peptostreptococcus micros, Fusobacterium nucleatum* from the orange complex, *Eubacterium nodatum* from the orange-associated complex, *Capnocytophaga gingivalis* from the green complex, and *Aggregatibacter actinomycetemcomitans.*

### 2.5. Statistical Analysis

The processing and statistical analyses of the obtained data were performed using BM SPSS Statistics v. 26 (SPSS INC., Chicago, IL, USA) software. All data were tested for parametric or non-parametric distribution. The minimum, maximum, mean, and standard deviation numbers were calculated. Analyzed quantitative variables were compared using Mann–Whitney U test and Spearman correlation, while for qualitative data, test Chi^2^ was used. Alterations between HS patients with a Hurley score from I to III and IHS4 mild, moderate, and severe were evaluated by Kruskal–Wallis 1-way analysis of variance on ranks. Effects of more than one independent variable on oral microflora were assessed with the use of Multivariate analysis of variance (MANOVA). A two-sided *p* value ≤0.05 was interpreted to indicate statistical significance.

## 3. Results

The study groups’ characteristics are presented in Table 1. A comparable age and gender structure was noticed in both groups. A remarkably higher number of active smokers was observed among HS individuals (*p* < 0.001).

**Figure 1:**
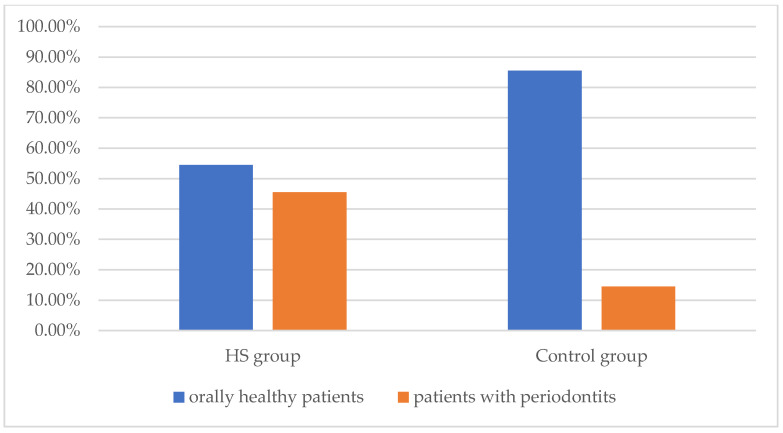
Prevalence of periodontitis in HS and control groups. *p* < 0.001, HS: hidradenitis suppurativa.

HS patients had a significantly higher prevalence of periodontitis than healthy controls (45.5% versus 14.5%, *p* < 0.001, Figure 1). The assessment of periodontitis severity in both groups is presented in Table 2. Grade C was the most frequent grade in HS individuals diagnosed with periodontitis (68%). In comparison, controls with periodontitis presented with grade A in most cases (50%). The most frequent stage in HS patients with periodontitis was stage II (40%), while among controls with periodontitis, stage III (50%) was most commonly diagnosed.

**Figure 2:**
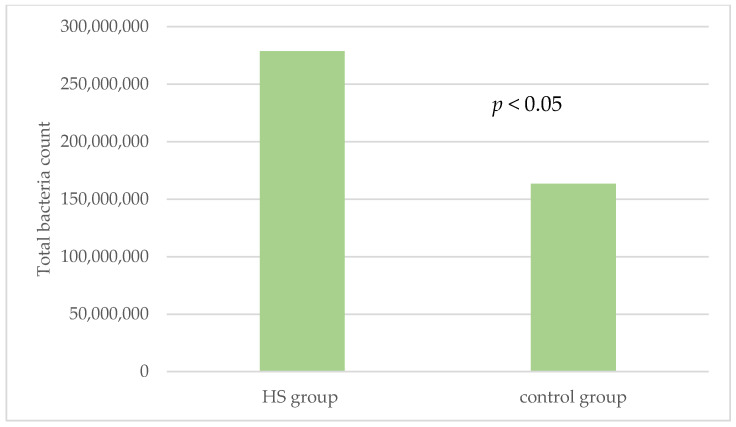
Average copy-counts number of total bacteria in the HS group and the control group. HS: hidradenitis suppurativa.

Figure 2 exposes the average copy-count numbers of total bacteria in both groups. The statistically significant higher values were found in HS patients (2.8 × 10^8^ average total bacteria count) compared to healthy controls (1.6 × 10^8^ average total bacteria count) (*p* < 0.05).

The majority of periopathogens tested were expressed at variable levels in the study and in the control group (Table 3). While *T. denticola*, *T. forsythia*, *P. micro*s, *F. nucleatum*, and *C. gingivalis* were more frequently isolated in patients with HS (*p* < 0.01), no significant difference was identified in *A. actinomycetemcomitans, P. gingivalis, P. intermedia*, and *E. nodatum* preponderance among the two studied groups (Table 3).

The most common bacteria detected in the HS group were *T. denticola* (70.9%), *C. gingivalis* (67.3%), *P. micros* (41.8%), and *T. forsythia* (40.0%), whereas among healthy controls, *C. gingivalis* (34.5%), *T. denticola* (20.0%), and *P. micros* (18.2%) were the most frequently isolated pathogens. Table 4 compares the prevalence of periodontal pathogens between smokers and non-smokers in both groups. Noteworthy, *T. denticola, T. forsythia*, and *P. micros* were statistically significantly more frequently found in smoking patients with HS (*p* < 0.01, Table 4). Interestingly, among smoking controls, *C. gingivalis* was more abundant (*p* < 0.05, Table 4). Importantly, MANOVA showed that smoking did not significantly influence the number of DNA copies of periopathogens or total bacteria count in both patients and controls. In addition, there was no correlation between total bacterial count as well as number of DNA copies of periopathogens and gender, body mass index, and education. Furthermore, HS severity assessed both with Hurley and IHS4 scales showed no relationship with copy numbers of total bacteria and quantity of periodontal pathogens (Table 5).

## 4. Discussion

In the present study, HS was associated with a higher prevalence of periodontitis compared with healthy subjects. Oral health may have an importance in the pathogenesis of many autoimmune skin conditions. Periodontal disease could play a role in the development and prognosis of psoriasis, pemphigoid, pemphigus, and lichen planus [14,27,28,29]. The more frequent prevalence of periodontitis in HS patients suggests that periodontal care and its impact on the oral microbiome might act as a modifiable risk factor for this entity.

Recent studies support the association between periodontitis and IMIDs, demonstrating the similarities in the imbalance of inflammatory cytokine network in these diseases. Th17 cells, their crucial cytokine interleukin 17 (IL-17), and interleukin 23 (IL-23) have been reported to play key roles in the pathogenesis of IMIDs as well as periodontitis [18,19].

IL-17 is a proinflammatory cytokine that is mainly derived from activated CD4+ helper T cells [30]. By promoting fibroblast upregulation of granulocyte colony-stimulating factor and CXC chemokines, this cytokine may influence bone marrow production and secretion of neutrophils and their chemotactic recruitment to the periodontal tissues [31]. In addition, IL-17 exhibits potent pro-osteoclastogenic capability that may also contribute to the development of periodontitis [32]. IL-17 increases receptor activator of nuclear factor kappa-Β ligand (RANKL) expression via osteoblasts, synovial cells, and mesenchymal cells [33]. Moreover, IL-17 may enhance the synthesis of matrix metalloproteinases in endothelial cells, epithelial cells, and fibroblasts, resulting in the destruction of connective tissue as well as the underlying bone [34].

IL-23 is a crucial cytokine involved in the differentiation and expansion of the Th17 subset [30]. Significantly higher tissue levels of IL-23 have been detected in periodontal lesions compared to control sites, suggesting that the IL-23/IL-17 axis plays a key role in the pathogenesis of periodontitis [35,36].

The IL-23/IL-17 signaling pathway also has pivotal importance in the development of chronic inflammation in HS [20,30,37]. IL-17 level has been shown to be significantly increased among HS patients and positively correlated with disorder severity [38,39]. The supplementary abundant expression of IL-23 by macrophages has been found in HS, indicating that the IL-23-induced Th17 pathways are involved in the disease process [20].

The majority of periodontopathic bacteria species were more frequently identified in the HS group in comparison to healthy controls. Importantly, *T. denticola*, which is one of the pivotal pathogens in periodontitis, was the most common isolated periopathogen among the HS group, affecting 70.9% of individuals. The total bacteria count and average number of copies of particular pathogens, such as *T. denticola*, *T. forsythia*, *P. micros,* and *F. nucleatum* tended to be higher in the HS group than in the control group.

The human microbiome may have major importance in autoimmunity. When self-tolerance mechanisms fail, residing microorganisms might elicit exaggerated immune responses [40]. The progression of periodontitis might be modulated by the interactions between periodontopathic microorganisms and host immunity. Periopathogens may damage the periodontum; nevertheless, an excessive and inappropriate host immune response stimulated by the bacteria might result in more severe and chronic destruction. Toll-like receptors (TLRs), which play an important role in the innate immune system via recognizing pathogen-associated molecular patterns, are involved in host innate immune responses to periopathogens and in the induction of adaptive immunity [21].

Recent studies provide evidence that HS may be associated with specific alterations in the cutaneous microbiome [4]. Various bacteria have been isolated from the abscesses and draining sinus tracts, including *Porphyromonas* species [41]. The increased expression of TLR2 at both the mRNA and the protein level has been reported in HS lesions compared with healthy skin [42]. Impaired Notch signaling, an essential part of HS pathogenesis, triggers the immune cascade and results in increased TLR- stimulated inflammatory responses [22].

Excessive immune activation due to microbial antigens observed in both periodontitis and HS may indicate a shared genetic predisposition and shared pathogenic pathways affecting dendritic cells and TLRs expression. Periopathogens can modulate IL-17 secretion by exploiting the resulting inflammatory milieu to access nutrients from tissue breakdown products as well as heme-containing molecules [32]. As IL-17 is one of the pivotal players in the pathogenesis of periodontitis and HS, increased expression of the cytokine might result in the progression of both diseases.

Apart from similar etiopathogenetic mechanisms, these entities share a common risk factor, which is smoking [43,44]. Smoking has been well-recognized as a risk factor for periodontitis progression [45]. Nevertheless, there are conflicting reports about the extent to which smoking influences the composition of the subgingival microbiome. Several investigations demonstrated that smoking patients with periodontitis have a higher prevalence and abundance of periodontitis-associated pathogens than non-smokers [46,47,48]. On the other hand, some studies could not corroborate those results [49,50]. In the present study, we revealed that mean DNA probe counts of pathogens species evaluated in current cigarette smokers did not significantly vary from those in non-smokers. Members of the red and orange complexes, including *T. denticola, T. forsythia,* and *P. micros,* were more prevalent among smoking patients with HS than in non-smoking patients from the study group. *C. gingivalis,* the member of the green complex, was more frequently found in smoking controls compared with non-smokers from the control group. The positive correlation between cigarette smoking and the higher prevalence of *T. denticola* and *P. micros* species has been previously reported [46]. Smoking is also a well-established risk factor for HS development [1,43]. It depletes normal commensal cutaneous microflora and may induce bacterial propagation and biofilm formation in HS lesions [51,52]. Biofilm harbored in sinus tracts can irreversibly attach to the epithelium, contributing to further inflammation [53].

This study has some limitations. First of all, the design of a descriptive cross-sectional study does not allow the detection of temporal interactions between HS and periodontal disease. Furthermore, the prevalence of HS is various, ranging from 0.00033 to 4.1% [54], and underdiagnosis or inadequate diagnosis is a common event, leading to difficulty in patient recruitment. This present research should be considered as a pilot study for further investigation of the association between HS and periodontitis. Therefore, the number of patients involved in our study (*n* = 55) seems to be appropriate, approximating that suggested to be optimal for this type of research [55].

## 5. Conclusions

To the best of our knowledge, this is the first study assessing oral health in patients with HS. In the present research, we revealed the higher prevalence of periodontitis and periodontitis-associated pathogens in HS patients compared to healthy controls. Therefore, a multidisciplinary interaction between dermatologists and periodontists is needed in HS individuals. HS management should include regular periodontal exams and appropriate treatment when required. Further studies on HS and periodontitis are crucial to shed light on potential mechanisms underlying the correlations between these conditions.

## Figures and Tables

**Table 1:** Patients’ characteristics.

	HS Group (*n* = 55)	Control Group (*n* = 55)
Age in years (mean ± SD)	36.22 ± 10.97 (19–61)	34.45 ± 10.29 (20–58)
Sex (Male, %) Smokers (%)	28 (50.91) 39 (70.91)	26 (47.27) 7 (12.73)

HS: hidradenitis suppurativa; SD: standard deviation.

**Table 2:** Staging and grading of periodontitis in HS and control groups.

	No (%) of Patients	
	HS Patients with Periodontitis (*n* = 25)	Controls with Periodontitis (*n* = 8)
Stage I	2 (8%)	0
Stage II	10 (40%)	3 (37.5%)
Stage III	7 (28%)	4 (50%)
Stage IV	6 (24%)	1 (12.5%)
Grade A	6 (24%)	4 (50%)
Grade B	2 (8%)	2 (25%)
Grade C	17 (68%)	2 (25%)

HS: hidradenitis suppurativa.

**Table 3:** Comparison of the copy number of pathogens collected from gingival sulci and the percentage of patients with particular bacteria in the HS group and the control group.

	Mean ± SD			No (%) of Infected Patients		
Periopathogens Tested	Subjects with HS	Control Group	*p*	Subjects with HS	Control Group	*p*
*A. actinomycetemcomitans*	2.7 × 10^2^ ± 1.5 × 10^3^	2.3 × 10^3^ ± 9.4 × 10^3^	0.332	4 (7.3%)	7(12.7%)	0.340
*P.gingivalis*	3.3 ×10^4^ ± 1.2 × 10^5^	2.7 × 10^4^ ± 1.3 × 10^5^	0.635	6 (10.9%)	8 (14.5%)	0.567
*T. denticola*	1.8 × 10^4^ ± 3.0 × 10^4^	8.3 × 10^3^ ± 3.1 × 10^4^	<0.001 *	39 (70.9%)	11 (20.0%)	<0.001 *
*T. forsythia*	7.0 × 10^5^ ± 5.0 × 10^6^	6.2 × 10^3^ ± 4.3 × 10^4^	0.001 *	22 (40.0%)	6 (10.9%)	<0.001 *
*P. intermedia*	2.7 × 10^4^ ± 9.9 × 10^4^	3.7 × 10^4^ ± 1.4 × 10^5^	0.381	12 (21.8%)	8 (14.5%)	0.323
*P. micros*	1.8 × 10^3^ ± 8.9 × 10^3^	1.0 × 10^3^ ± 4.5 × 10^3^	0.010 *	23 (41.8%)	10 (18.2%)	0.007 *
*F. nucleatum*	5.9 × 10^2^ ± 3.0 × 10^3^	10 ± 55	0.007 *	11 (20.0%)	2 (3.6%)	0.008 *
*E. nodatum*	1.5 × 10^3^ ± 1.1 × 10^4^	54 ± 3.1 × 10^2^	0.625	3 (5.5%)	2 2 (3.6%)	0.647
*C. gingivalis*	2.9 × 10^3^ ± 8.0 × 10^3^	3.8 × 10^3^ ± 1.2 × 10^4^	0.003 *	37 (67.3%)	19 (34.5%)	0.001 *
Total bacteria count	2.8 × 10^8^ ± 4.3 × 10^8^	1.6 × 10^8^ ± 2.6 × 10^8^	0.023 *	55(100%)	55(100%)	NA

* *p* < 0.05, statistically significant differences between groups, HS: hidradenitis suppurativa, SD: standard deviation.

**Table 4:** Comparison of the prevalence of periodontal pathogens among smokers and non-smokers.

	HS Group			Control Group		
Periopathogens Tested	No (%) of Infected Smokers (*n* = 39)	No (%) of Infected Non-smokers (*n* = 16)	*p*	No (%) of Infected Smokers (*n* = 7)	No (%) of Infected Non-smokers (*n* = 48)	*p*
*A. actinomycetemcomitans*	3 (7.69) %	1 (6.25) %	NS	0%	7 (14.58) %	NS
*P.gingivalis*	5 (12.82) %	1 (6.25) %	NS	2 (28.57) %	6 (12.5) %	NS
*T. denticola*	31 (79.49) %	8 (50.0) %	0.029	3 (42.85) %	8 (16.67) %	NS
*T. forsythia*	19 (48.72) %	3 (18.75) %	0.039	1 (14.29) %	5 (10.42) %	NS
*P. intermedia*	10 (25.64) %	2 (12.5) %	NS	1 (14.29) %	7 (14.58) %	NS
*P. micros*	21 (53.85) %	2 (12.5) %	0.005	2 (28.57) %	8 (16.67) %	NS
*F. nucleatum*	7 (17.95) %	4 (25.0) %	NS	1 (14.29) %	1 (2.08) %	NS
*E. nodatum*	1 (2.56) %	2 (12.5) %	NS	1 (14.29) %	1 (2.08) %	NS
*C. gingivalis*	23 (58.97) %	14 (87.5) %	NS	5 (71.43) %	14 (29.17) %	0.028

*p* < 0.05, statistically significant differences between groups, HS: hidradenitis suppurativa, NS*:* no statistically significant difference.

**Table 5:** Spearman correlation coefficient (r_S_) between Hurley and IHS4 scores and DNA copy number of periopathogens and total bacteria.

Periopathogens Tested		Hurley Score	IHS4
*A. actinomycetemcomitans*	r_S_	0.024	0.058
	*p*	0.866	0.685
*P.gingivalis*	r_S_	0.116	0.076
	*p*	0.412	0.596
*T. denticola*	r_S_	0.230	0.211
	*p*	0.101	0.136
*T. forsythia*	r_S_	0.257	0.212
	*p*	0.066	0.135
*P. intermedia*	r_S_	−0.057	−0.090
	*p*	0.686	0.530
*P. micros*	r_S_	0.229	0.162
	*p*	0.102	0.257
*F. nucleatum*	r_S_	−0.049	0.039
	*p*	0.729	0.784
*E. nodatum*	r_S_	0.152	−0.026
	*p*	0.282	0.854
*C. gingivalis*	r_S_	−0.199	−0.186
	*p*	0.158	0.191
Total bacteria count	r_S_	0.030	−0.025
	*p*	0.833	0.861

IHS4: International Hidradenitis Suppurativa Severity Score System.

## Data Availability

The datasets generated and analyzed in the current study are available from the corresponding author upon reasonable request.
